# Increasing fibre intake in the UK: lessons from the Danish Whole Grain Partnership

**DOI:** 10.1017/S0007114523002106

**Published:** 2024-02-28

**Authors:** Neil Bernard Boyle, Katie Adolphus, Samantha J. Caton, Fiona C. Croden, Louise Dye, Amy Glass, Kate Halliwell, Gitte L. Hansen, Lotte Holm, Peter Jackson, Fiyin Makinwa, Bente Stærk, Nicholas Wilkinson

**Affiliations:** 1 School of Psychology/School of Food Science & Nutrition, University of Leeds, Woodhouse Lane, Leeds, LS2 9JT, UK; 2 School of Health and Related Research, Public Health, University of Sheffield, Sheffield, UK; 3 Food and Drink Federation, London, UK; 4 Danish Cancer Society, Strandboulevarden 49, DK-2100 Copenhagen, Denmark; 5 Department of Food and Resource Economics, University of Copenhagen, Frederiksberg C, Denmark; 6 Department of Geography, University of Sheffield, Sheffield, UK; 7 Danish Veterinary and Food Administration, Stationsparken 31-33, DK-2600 Copenhagen, Denmark

**Keywords:** Fibre, Whole grain, Danish Whole Grain Partnership, Dietary intervention

## Abstract

Diets deficient in fibre are reported globally. The associated health risks of insufficient dietary fibre are sufficiently grave to necessitate large-scale interventions to increase population intake levels. The Danish Whole Grain Partnership (DWP) is a public–private enterprise model that successfully augmented whole-grain intake in the Danish population. The potential transferability of the DWP model to Slovenia, Romania and Bosnia-Herzegovina has recently been explored. Here, we outline the feasibility of adopting the approach in the UK. Drawing on the collaborative experience of DWP partners, academics from the Healthy Soil, Healthy Food, Healthy People (H3) project and food industry representatives (Food and Drink Federation), this article examines the transferability of the DWP approach to increase whole grain and/or fibre intake in the UK. Specific consideration is given to the UK’s political, regulatory and socio-economic context. We note key political, regulatory, social and cultural challenges to transferring the success of DWP to the UK, highlighting the particular challenge of increasing fibre consumption among low socio-economic status groups – which were also most resistant to interventions in Denmark. Wholesale transfer of the DWP model to the UK is considered unlikely given the absence of the key ‘success factors’ present in Denmark. However, the DWP provides a template against which a UK-centric approach can be developed. In the absence of a clear regulatory context for whole grain in the UK, fibre should be prioritised and public–private partnerships supported to increase the availability and acceptability of fibre-rich foods.

In all regions of the globe, dietary fibre is consumed in amounts below recommended levels^(1)^. In 2019, a diet low in fibre was a risk factor contributing to an estimated 15·3 million disability-adjusted life years (95 % uncertainty interval 9·11–22·0 million) and 606 000 deaths (95 % uncertainty interval 342 000–887 000^(2)^). In the UK, in 2015 the Scientific Advisory Committee on Nutrition (SACN^(3)^) recommended the greatest level of health benefit from fibre requires a daily intake of 30 g/d for adults (measured using the Association of Official Analytical Chemists’ method^(4)^.^1^ On average, all UK population age groups fall approximately one-third short of this recommended daily intake, with only 9 % of adults aged 19–64 years, and 6 % of adults over 65 years meeting the recommended intake levels (National Diet and Nutrition Survey^(5)^); comparable proportional shortfalls are evident across all age groups (see Fig. 1).

**Fig. 1.**
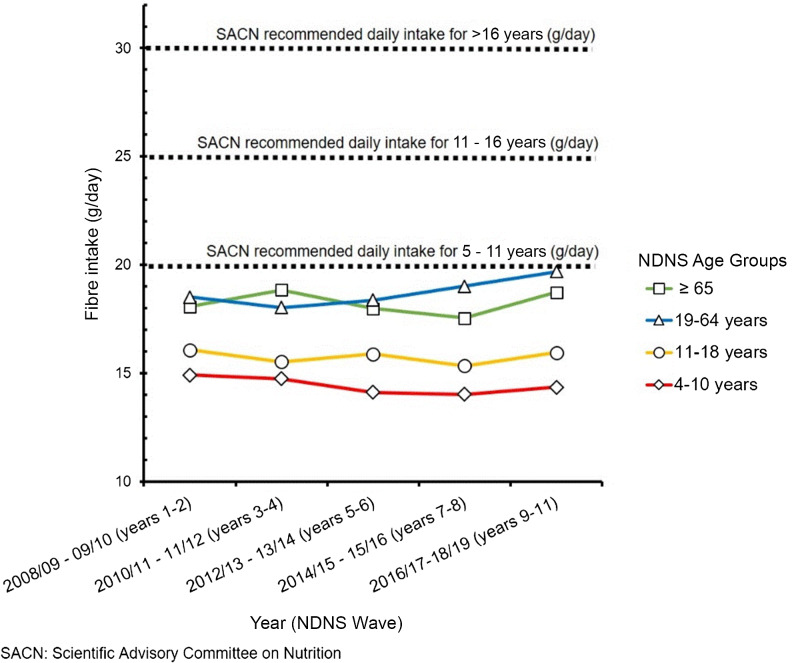
Mean UK daily fibre intake from National Diet and Nutrition Survey rolling programme 2008–2009 to 2016–2017 (Waves 1–2 to 9–11) by age group. SACN (2015) recommended daily fibre intake values per age group shown by broken reference lines. Amended from: Public Health England, NDNS available at: https://www.gov.uk/government/statistics/ndns-results-from-years-9-to-11–2016-to-2017-and-2018-to-2019.

Dietary modelling of the feasibility of meeting the SACN 30 g/d fibre recommendation – while adhering to other dietary guidelines such as the Eatwell Guide – demonstrated that it is possible to consume 30 g of fibre a day if all meals are based on starchy foods (mainly whole-grain options and potatoes with skins), and approximately 8 portions of fruit and vegetables and high-fibre snacks are consumed daily^(6)^. Whilst the SACN fibre recommendation is feasible, the required dietary pattern is not reflective of average diets in the UK and would require substantial change in dietary habits^(6)^. Achieving the SACN 30 g/d fibre recommendation is therefore a considerable challenge requiring collaboration with a number of stakeholders including the food industry, health professionals and academics.

The picture is not universally bleak. Our European partners in Denmark have made impressive gains in fibre-rich food intake levels by reversing the national downward trend of whole-grain consumption. The Danish Whole Grain Partnership (DWP; https://fuldkorn.dk/english/) is a collaborative public–private enterprise model that has brought together government, health NGO and the food industry to work collaboratively to increase intakes of whole grains in the Danish population – a feat that is mutually beneficial to all partners. In the UK, low intake of whole grains has been identified as the leading risk factor for diet-related ill health (specifically CVD-related deaths and disability-adjusted life years^(7)^). Therefore, replicating the impacts of the DWP could have a significant positive impact on diet-related health in the UK population. However, there are significant challenges and barriers to the transferability of the DWP approach to the UK that need to be considered and mitigated. Based on the discussions of a working group comprising primary partners of the DWP (representatives of the Danish Cancer Society and the Danish Veterinary and Food Administration), academics from the H3 project (https://h3.ac.uk/) and colleagues from the UK Food and Drink Federation, this article explores the potential transferability of the experience and approaches of the DWP to the UK. This article considers the factors contributing to the success of the DWP in achieving significantly increased consumption of whole grains and relates these learnings to the UK context with specific consideration given to political, regulatory and socio-cultural factors.

## The Danish Whole Grain Partnership

**Fig. 2.**
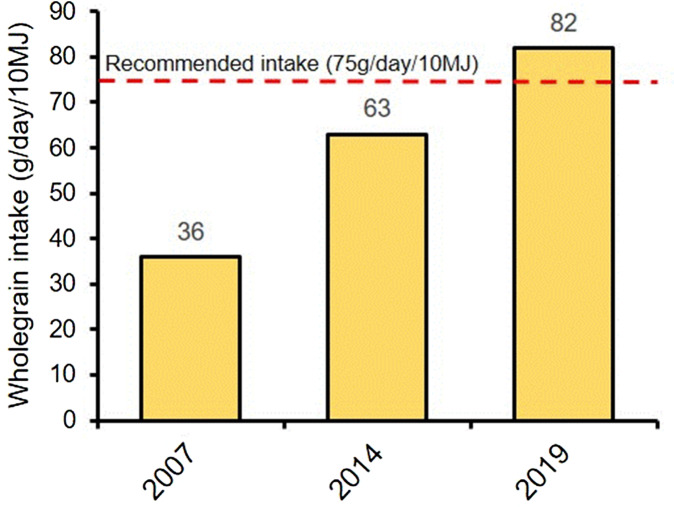
Danish wholegrain intake/d (g/10 MJ) 2007–2019. Data taken from Mejborn. Danskernes fuldkornsindtag 2011–2013 (Intake of wholegrain in Danish population 2011–2013). 2014: Lyngby; and Andersen *et al.* (2021). Intake of whole grain and associations with lifestyle and demographics: a cross-sectional study based on the Danish Diet, Cancer and Health—Next Generations cohort. *European Journal of Nutrition*, 60(2), pp. 883–895. doi: 10·1007/s00394–020–02289-y.

A public–private partnership was established in 2008 to promote whole-grain intake in the Danish population. The aim was to increase availability of whole-grain products in the market and to raise awareness of the health benefits of whole grain. Since the formation of the DWP, intake of whole grain in the Danish population has increased substantially (see Fig. 2), a development which is widely ascribed to the activities of the partnership. In 2019, the EU commission awarded the DWP ‘best practice certificate reaching Sustainable Development Goals (SDG)’ to promote population health, and the Organisation for Economic Co-operation and Development recently named it a best practice case in how to transfer an intervention^(8)^.

The DWP was established in recognition that food system challenges could be more efficiently tackled by collaborative enterprise. During the 1990s and 2000s, Danish public authorities recorded a decline in consumption of traditional rye bread, which increased concerns about the associated health consequences of reduced intake^(9)^. In 2008, the Danish Cancer Society, informed by emerging evidence of a relationship between whole-grain intake and cancer^(10)^, focused their health promotion efforts on whole-grain consumption. At the same time, the Danish bread industry feared the growing popularity of modern, fat-rich low-carb diet food trends – such as the Atkins diet – would threaten an already declining bread market^(11)^. On this basis, the Danish Veterinary and Food Administration, The Danish Cancer Society and the Danish Food and Drink Federation joined forces to develop the DWP. By 2008, the partnership included fourteen partners across different public and private sectors.

### Process and strategy

The primary developmental step of the DWP involved the partners developing a shared knowledge base. This included an agreed definition of whole grain, a review of the evidence for health benefits of whole grains^(12)^ and an ethnographic study of consumer knowledge and perceptions of whole grains^(13)^. On this basis, the Danish Veterinary and Food Administration amended the dietary guidelines to include a recommended intake for whole grain^(9)^. An agreed strategy was subsequently adopted that targeted both the demand and supply of whole grain. It included public information and campaign activities to inform the population of the benefits of whole grains, and activities to help shape new norms for whole-grain consumption via campaigns, events and structural changes. The introduction of a standardised whole-grain logo was central to the DWP approach. The whole-grain logo was developed and promoted as a way to communicate nutritional advice on behalf of Danish authorities to help consumers recognise whole-grain products. Food manufacturers can put the whole-grain logo on their products provided they fulfil specified criteria that stipulate the content of whole grain for defined product categories^(14)^. Products are also required to fulfil the nutrient profile in accordance with the Nordic Keyhole nutrition label^(15)^; this prevents the logo being used on unhealthy products, such as high fat/sugar biscuits or cakes. For industry, the logo represents a competitive advantage and serves as an incentive to produce products aligned with the DWP’s strategic aims of increasing the availability of whole-grain products, developing new whole-grain products and incorporating whole grains in all cereal-based products. The logo has been incorporated into the ordinary food labelling control system in Denmark^(9)^.

DWP activities and outcomes are consistently monitored and evaluated. Ambitious and exact incremental goals for strategic outcomes have been regularly set up addressing the level and demographic distribution of whole-grain intake in the Danish population, logo awareness in the population and number of whole-grain logo-labelled products on the market.

### Danish Whole Grain Partnership organisation

The DWP has a formal structure, comprising a board of representatives from each partner category (government, health NGO and industry) and a professional secretariat. The board decides on strategy, action plans, budgets and partnership financing. All partners are responsible for executing activities; partners finance all activities.

Each partner category plays distinct and complementary roles in the partnership. For example, the logo and the criteria for its use were created by a joint effort of the partners. Public authorities enforce the logo, and they issue dietary guidelines, educate the public about the importance of whole grains for health and develop guidelines for relevant professionals. The food industry partners (millers, craft bakeries and food manufacturers) increase the supply of whole-grain products meeting the logo criteria and reformulate existing products to increase whole-grain content. The retail sector promotes whole grain through in-store activities and special deals. Health NGO communicate the importance of whole grains for health and add to the evidence base by funding clinical and epidemiological research.

### Danish Whole Grain Partnership success factors

**Fig. 3.**
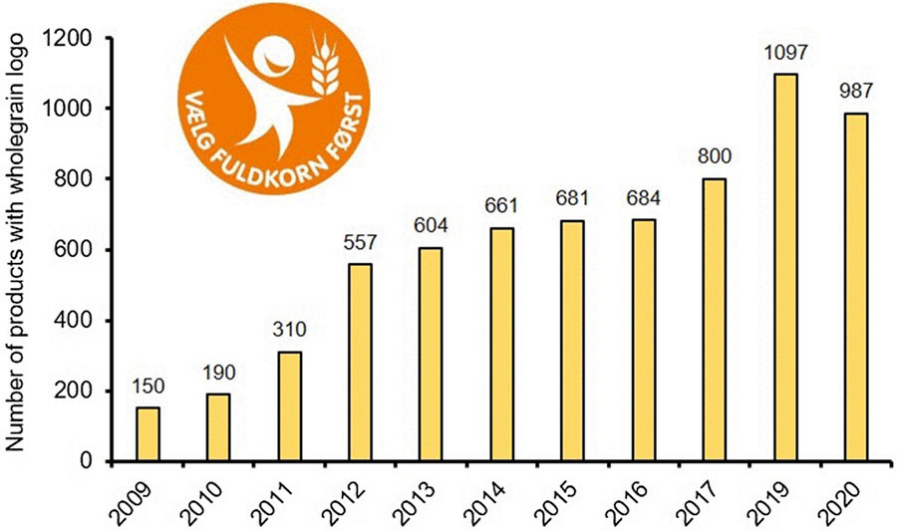
Danish market availability of food products branded with the DWP wholegrain logo 2009–2020. Source: Data from the Danish Whole Grain Partnership: https://fuldkornet/.dk/om-partnerskabet/fuldkornslogo.

Whole-grain intake and availability of whole-grain products on the market have increased considerably in Denmark since the establishment of the DWP (see Fig. 2 and 3). By 2019, the daily intake of whole grain in the Danish population rose by 128 % (from 36 to 82 g/MJ) and the share of the population eating the recommended amount of whole grain per day rose from 6 % to 54 %^(10,16)^. Up to 2014, children’s intake rose by 118 % (from 28 to 58 g/MJ), and among the quarter of the population with the lowest whole-grain consumption, intake doubled (from 12 to 24 g/MJ)^(16)^.

Several factors may account for the success of the DWP. The partnership built upon a previous successful public–private collaboration that promoted ‘six-a-day’ fruit and vegetable consumption in Denmark^(11)^. This existing partnership experience fostered trust between actors across the different societal sectors. The number of industrial partners in the DWP is currently 29, which emphasises the attractiveness to the food industry of being able to use the whole-grain logo and engage with the activities of the DWP. For the general population, the partnership’s focus on whole-grain resonates with Danish history and tradition. Since the middle ages, rye bread has been one of the most important sources of nutrition in Denmark^(17)^, and even today, rye bread is seen as a daily bread, a filling bread and source of energy fuelling the body for work^(13)^. Nevertheless, since 1955, rye bread consumption decreased in Denmark while consumption of wheat bread increased^(18)^. Since the 1990s, the dwindling popularity of traditional Danish whole-grain food has been part of public discourse and acknowledged as an unfortunate development for public health. This provided an existing platform and public awareness base on which the DWP could build. Further, an important part of building public awareness of the benefits of consuming whole-grain food was the representation of governmental food authorities in the partnership. Historically, Danish food-based dietary guidelines encouraged the population to eat ‘coarse bread’. In accordance with the agreed DWP evidence base, the wording of recommendations was changed to ‘Eat whole-grain bread’^(11)^. This ensured that messages from the partnership and the general communication and information activities of food authorities became aligned with the strategic aims of the DWP.

## Transferability of the Danish Whole Grain Partnership to the UK

The Organisation for Economic Co-operation and Development Healthy Eating and Active Lifestyles: Best Practices in Public Health Report^(8)^ assessed the transferability of the DWP to other member nations based on three transferability context indicators for translational success: *sector-specific context, political context* and *economic context*. The UK was given the highest transferability rating across all indicators (see Table 1 for summary of transferability indicators for the UK). Whilst this suggests the UK’s general political economy landscape is well suited to the introduction of population-level public health interventions such as the DWP, there are a number of significant barriers and contextual differences that need to be considered when assessing the transferability of the DWP model to the UK.

**Table 1.** Organisation for Economic Co-operation and Development (OECD) transferability indicator summary for transferability of the DWP to the UK

OECD transferability indicator	Indicator definition	UK transferability status
Sector-specific context	Existing structure in place to support front-of-pack nutrition labelling and nutrition and health claims on fibre (e.g. regulatory frameworks)	UK has well-established front-of-pack nutrition labelling scheme; however, this indexes overall nutrition quality based on salt, sugar and fat content and does not currently reflect whole grain or fibre content. Following the UK’s departure from the EU on 31 January 2020, the UK has adopted the original Nutrition and Health claims regulation (Regulation (EC) No. 1924/2006) and the UK authorisation process by the newly formed UK Nutrition and Health Claims Committee process is similar to the original EFSA substantiation process. The EU Register of authorised claims was adopted by the UK for use in Great Britain and hence, there are authorised health and nutrition claims on fibre (but not whole grain) for use in all commercial communications
Political context	Level of political prioritisation of healthy eating	The UK has a number of large-scale national action plans that target a reduction in unhealthy eating (e.g. Eat Well Guide, ‘5 A Day’ campaign). Furthermore, the 2021 Food (Promotion and Placement) Regulations in England aims to restrict the placement and in-store promotions of prepacked foods that are high in fat, sugar and salt as defined by the UK nutrient profiling model. The model provides a single score for any food product, based on calculating the number of points for ‘negative’ nutrients which can be offset by points for ‘positive’ nutrients (including fibre). The recent Henry Dimbleby independent review of the UK food system firmly placed unhealthy eating at the heart of recommendations for improving the food landscape in the UK, introducing the term ‘junk food cycle’. However, the Government’s initial response to the review has largely ignored or deferred many of the more contentious recommendation on unhealthy eating
Economic context	Proportion of health spending allocated to prevention campaigns	The UK has one of the highest prevention expenditures as a percentage of current health expenditure (CHE) of OECD members, indicating a high priority given to preventative health interventions^(8)^

### Political and legislative factors

Geographically, the UK is significantly bigger than Denmark (UK: 243 610 km^2^ *v*. Denmark: 42 920 km^2(19)^) and has a substantially larger population (UK: approximately 68 million *v*. Denmark: approximately 6 million^(20)^). These differences inherently increase the complexity of affecting change due to the need to reach a greater number of people across a larger, more geographically diverse area. Further, the UK is made up of four nations, and policy responsibility, including legislative powers, in certain areas is devolved to the individual nations. This includes the overwhelming majority of food policy and regulations. Danish Government backing for the DWP, in concert with health NGO and industry, was instrumental to its success in the production of consistent, authority-endorsed definitions and recommendations for whole-grain intake. This same commitment is likely more challenging in the UK due to the devolved nature of government. For a successful partnership in the UK, all four nations would need to be involved in the design and delivery of the initiative. This situation is complicated as the competencies for different aspects of food policy sit within different government departments within each nation.

Devolution across dietary policy can result in different policies and priorities for the nations. For example, the recently published Government Food Strategy for England sets out an intention for ‘government and industry working in partnership on a shared endeavour to promote healthier diets’. This is a positive signal towards a public–private partnership approach; however, as this strategy is for England, it is unclear whether the other nations share this vision for partnership with industry. Each nation also has a separate dietary and obesity strategy that sets out their priorities and policies to improve diet. The situation has been further complicated post-Brexit wherein the Westminster government is currently deciding which parts of EU legislation, previously set in Brussels, should be retained within the UK and which should be subject to new domestic legislation. This has already been reflected in heated debates in Parliament about chlorinated chicken, the regulation of antimicrobials and the use of neonicotinoids in pesticides^(21,22)^. Despite this increased complexity, collaborative working across the nations on unified public policy is possible as previously demonstrated by joint consultation for front-of-pack nutrition labelling^(23)^ and the fortification of flour with folic acid^(24)^. However, there is currently little to suggest whole grain, or indeed fibre in general, is high on the political agenda in the UK (whole grain is absent from the Government Food Strategy; fibre is mentioned once in relation to low intake in deprived groups).

### Regulatory factors

The UK Nutrition and Health claims regulation presents both challenges and opportunities for transferability of the DWP to the UK. The regulation applies to all nutrition and health claims made in commercial communications, whether in the labelling, presentation or advertising of foods to the consumer. Following departure from the EU, the UK adopted the original regulation (Regulation (EC) No. 1924/2006) and the EU Register of authorised nutrition and health claims for use in Great Britain. Furthermore, a new committee, the UK Nutrition and Health Claims Committee (UKNHCC), assumed responsibility for the assessment of the scientific evidence in support of submitted new claims in Great Britain. UKNHCC operates in a similar way to the European Food Safety Authority (EFSA) and intends to have similar timescales and evidence evaluation process. Dossiers submitted in the UK for a new health claim based on new studies and/or proprietary data (i.e. unpublished results not in the public domain and owned by the food manufacturer) undergo a full scientific assessment by UKNHCC, taking account of the totality of the available scientific data, the characterisation of the food or constituent and weighing the evidence provided in the applicant’s dossier. Hence, the newly formed UKNHCC, and separate assessment process to EU, opens potential for new claims opportunities in the UK. This could stimulate food manufacturers to reformulate or produce new products high in specific active dietary fibres with associated proprietary evidence to pursue exclusive use of health claims as a competitive advantage; incentivising reformulation/development of new whole-grain products on the market was key to the success of the DWP.

Under the nutrition and health claims regulation, there are authorised nutrition and health claims on fibre; however, none on whole grain given the lack of definition. A range of health claims relating to specific fibre types have been authorised for use in England, which describe the relationship between consumption of the fibre type and health. No health claims have been approved for ‘dietary fibre’ and ‘whole grain’ as both terms are not sufficiently characterised for a scientific assessment. However, claims on specific fibres have received favourable opinions and been approved in relation to an increase in faecal bulk, reduction in intestinal transit time, reduction in post-prandial glycaemic responses and maintenance of normal blood cholesterol concentrations. A full list of authorised claims can be found in the Great Britain nutrition and health claims register (https://www.gov.uk/government/publications/great-britain-nutrition-and-health-claims-nhc-register). Whilst the authorised nutrition and health claims on fibre are not entirely compelling for consumers, they may incentivise the food industry to reformulate existing products or produce new products to meet the approved nutrition and health claims’ conditions of use and may provide a competitive advantage in the market. Moreover, nutrition and health claims on pack or in any advertising of foods to the consumer may help drive dietary changes and increase awareness of fibre in consumers.

A critical barrier to transferring the DWP to the UK lies in the divergent definitions and dietary guidelines related to whole grain. A primary step of the DWP was to clarify the definition of whole grain and develop a dietary recommendation of 75 g whole grains/d (equivalent to four portions/d)^(12)^. Having a recommendation enabled clear messaging and subsequent monitoring of the success of the programme. The UK does not currently have a standardised definition of whole grain, nor a recommended whole-grain intake amount^(25)^. A clear, precise food authority-endorsed whole-grain logo was crucial to the success of the DWP. At present, without a definition or dietary recommendation for whole grain in the UK, this may be challenging to transfer. This significantly impedes capacity to employ whole grains as an anchor for any UK partnership. First, the current lack of definitions and guidelines would preclude establishment of an agreed and coherent knowledge base. Second, this lack of clarity fundamentally renders any attempt to introduce a standardised whole-grain logo redundant. The official Danish whole-grain logo was useful in incentivising industry to participate in the Danish public–private partnership and reformulate their products in line with nutritional guidelines and gain credit for their actions. However, nutrition and health claims may not provide the same incentive in the UK.

Despite the lack of government definition of whole grain in the UK, a number of attempts to define whole grain have been proposed. The Institute of Grocery Distribution (IGD) developed a guidance document in 2007 aimed at retailers and manufacturers on a UK-relevant whole-grain definition, recommended levels of whole-grain inclusion and how to communicate this to consumers^(26)^. The IGD definition and international definitions of whole grain share common characteristics, for example, cereal grains containing endosperm, bran and germ in their original proportions. More recently, a Whole Grain Initiative working group of academics and food industry representatives convened to agree a definition of whole grain as an ingredient and what constitutes a whole-grain food^(27)^. The Healthgrain Forum aimed to provide a scientifically meaningful definition that would be both useful to industry and permitting informative food labelling to increase consumer understanding and acceptance. A whole grain is defined as: ‘*the intact, ground, cracked, flaked or otherwise processed kernel after the removal of inedible parts such as the hull and husk. All anatomical components, including the endosperm, germ, and bran must be present in the same relative proportions as in the intact kernel*’^(28)^ (p. 3). A whole-grain food should contain at least 50 % whole-grain ingredients based on dry weight. Foods containing a minimum of 25 % whole-grain ingredients by dry weight can make a front of pack label claim, but the product name should not designate the product as ‘whole grain’^(28)^. It is also recommended that whole-grain foods should meet the accepted local nutritional standards for healthy foods, an approach employed by the DWP using the Nordic Keyhole standards as a benchmark. The acceptance and adoption of these definitions would greatly increase the efficacy of attempts to increase the availability and acceptance of whole grain and whole-grain foods. Particularly since there is a currently a distinct lack of clarity and consistent regulation for the minimum whole-grain content necessary for a food product to be labelled and promoted as a whole-grain food. However, given what to date has been a lack of UK government support or enthusiasm for whole-grain definitions or recommended intakes, it is difficult to see how a public–private partnership on whole grain could work.

Given such barriers, an alternative approach is to consider fibre, where there are definitions and dietary recommendations and which is still strongly linked to health outcomes. Whole grains and fibre are often discussed simultaneously, possibly due to evidence that fibre is a crucial factor in the health benefits of whole-grain intake^(3)^. A rising number of national dietary guidelines, including the UK, also recommend whole-grain foods as a good source of fibre. Indeed, low intake of whole-grain foods in the UK is a likely contributory factor to inadequate fibre intake^(25)^. Naturally, the two are distinct in that fibre is a nutrient, and whole grain is a food group that provides fibre – as well as other important nutrients. However, an increase in whole-grain consumption would increase fibre intake; conversely, any increase in fibre intake can promote the intake of whole grains. The SACN report on Carbohydrates and Health^(3)^ did not find sufficient evidence to develop a dietary recommendation for whole grain but did propose increasing the dietary recommendation for fibre from 24 g to 30 g/d for adults, which the government adopted. There is also a clearer definition of fibre – albeit not universally accepted or adopted – which enable companies to understand how to produce higher fibre products. Fibre is defined in the Food Information Regulation^(29)^ and covers all carbohydrate polymers with three or more monomeric units that are neither digested nor absorbed in the small intestine and are: (1) naturally occurring edible carbohydrate polymers in food or (2) edible carbohydrate polymers, synthetic or obtained from food raw ingredients, which have a beneficial physiological effect demonstrated by generally accepted scientific evidence.

As well as a clear definition and a dietary recommendation, there is also legislation that sets out how fibre can be declared on the label, both within the ingredients declaration, but perhaps more importantly as a claim on the front of pack (a nutrition claim of ‘source of’ or ‘high in’ fibre can be made if food meets nutrition thresholds 3 g/100 g or 1·5 kcal/100 kcal and 6 g/100 g or 3 g/100 kcal, respectively^(30,31)^). Taken together, this provides food companies clarity on how to increase the availability and communication of higher fibre options for consumers, compared with an approach focusing solely on whole grains. Food manufacturers might be encouraged to produce higher fibre foods for a variety of reasons, from enabling products to have a claim on pack to achieving fat and sugar reduction using functional fibres.

Fibre is also part of the UK Nutrient Profiling Model, originally developed by the Food Standards Agency (FSA) in 2004–2005 to provide Ofcom, the broadcast regulator, with a tool to differentiate foods based on their nutritional composition in the context of television advertising foods to children. The model uses a simple scoring system where points are allocated on the basis of the nutrient content of 100 g of a food or drink. Points are awarded for ‘A’ nutrients (energy, saturated fat, total sugar and Na), and for ‘C’ nutrients (fruit, vegetables and nut content, *fibre* and protein). The score for ‘C’ nutrients is then subtracted from the score for ‘A’ nutrients to give the final nutrient profile score. Foods scoring four or more points, and drinks scoring one or more points, are classified as ‘less healthy or ‘high in fat, sugar and salt’. There is evidence to support the integration of whole grain into nutrient profile models^(32,33)^, but as yet, there are no plans to implement this.

The UK Nutrient Profiling Model also underpins the 2021 Food (Promotion and Placement) Regulations in England, a series of restrictions on the promotion and placement of pre-packaged high in fat, sugar and salt foods. The new regulations affect medium and large (with 50 employees or more) retailers, manufacturers and food business operators. In practical terms, the new policy means that many brands will need to reformulate if they want to avoid the volume and placement restrictions. Companies may be reformulating based on these new regulations to make products non-high in fat, sugar and salt by adding fibre as one aspect of this reformulation and offers a common purpose among stakeholders for a fibre/whole grain focused UK public–private partnership.

In 2020, Department of Health and Social Care (DHSC) launched a four-nation evidence review on front-of-pack nutrition labelling. This sought views on a variety of things including whether labelling should reflect dietary advice on fibre. Currently, front-of-pack nutrition labelling in the UK is focused only on nutrients of concern. The inclusion of fibre may help to raise awareness of fibre and dietary sources and drive reformulation to increase fibre so that this can be highlighted on the label. If taken forward, how fibre is displayed on the food packaging would need to be trialled and tested to ensure consumer understanding. As food is freely traded within Great Britain, it is important that any approach to labelling spans the three nations to avoid consumer confusion. Currently, food labelling in Northern Ireland needs to align with the EU; it would also be beneficial if any approach developed in Great Britain is also compliant with EU legislation to help ensure free flow of goods within the UK and into Europe and beyond. The newly agreed Windsor Framework presents an evolving situation that will change the current practice.

The criteria used to underpin any logo, whether this is solely based on fibre/whole grain, for the UK would need to be carefully considered. Currently, there are various schemes to define healthiness of food and drink products – the 2004–2005 UK Nutrient Profiling Model, Traffic Light labelling thresholds, Better Health Good Choice criteria and Government reformulation targets. Information to consumers provided on pack is also increasing, including animal welfare schemes, nutrition and health claims, and more recently, eco labelling. How consumers react to and understand on pack information is important and the addition of more logos, like the logo used in the DWP, could complicate this. Before the addition of a logo, a review of consumer understanding is essential and any criteria must be evidence-based and developed in consultation with industry.

An important aspect of a public–private partnership is public health messaging and how consumers respond to this. In the UK, this has previously been clearly demonstrated with salt reduction. Public health messaging to support industry action in salt reduction was instrumental in raising consumer awareness about the health implications of a diet high in salt. This successfully reduced UK salt intakes by 11 % in the last decade^(34)^. However, there has been a lack of public health messaging around fibre, despite the increase in dietary intake recommendation in 2015. As a result, there is low consumer awareness of the dietary recommendations, sources and benefits of a diet high in fibre. Polling shows that consumers are not aware of the wide-ranging benefits of fibre – outside of bowel health – or that fibre is found in a wide range of foods, not just brown and wholemeal carbohydrates^(35,36)^. This has obvious implications for anchoring public health messages and campaigns to increase the acceptability of high-fibre foods and underlies the need for significant increases in public awareness of fibre and the benefits of its consumption as a key element of any partnership intervention.

Considering the challenges identified, fibre may currently be a more relevant focus for a public–private partnership in the UK. The food industry has already made efforts in this space, for example, the Food and Drink Federation recently launched a new initiative called Action on Fibre^(35)^. Companies signed up to this initiative are committed to help bridge the gap between fibre intakes and dietary recommendations through various approaches: from reformulation to increase fibre in products, to marketing and labelling to raise consumer awareness, and working with the supply chain and employees to encourage increases in fibre consumption. This does not discount a focus on whole grain in the future. In a recent SACN horizon scanning meeting (17th June 2022, London), the Committee agreed to add whole grain to their work programme, with a first step to develop an overview and initial assessment of existing definitions on whole grain. This may indicate a potential appetite to address regulatory barriers to whole-grain promotion and could signal future developments in the UK in relation to whole-grain definitions and recommendations.

### Socio-cultural factors

Consumption of whole grains is embedded in Danish culture in a way not replicated in the UK. Rye is the largest contributor to the whole-grain intake for both Danish children and adults. This is culturally tied to the traditional consumption of open-face rye bread sandwiches at lunchtimes. Oats (including porridge) contribute the second largest source of whole grain, largely associated with breakfast habits^(12)^. Eating rye bread has been an unbroken dietary staple for centuries in Denmark. Indeed, a primary catalyst for the formation of the DWP was to reverse a prolonged decline in the consumption of traditional whole-grain produce, in part, by promoting a return to traditional Danish foods. Contrastingly, there are no commonly consumed whole-grain foods that form a key part of traditional UK diets. The widespread consumption of whole grain has been largely absent in the UK since the 18th century as the Second Agricultural and the Green Revolutions shifted the processing of grain from coarse unrefined flour to large-scale production of refined flour. Culturally, the consumption of refined flour was also aligned with social position, with refined grains associated with higher-class status^(37)^. Paradoxically, whole and unrefined grain products are now often considered aspirational, associated with the increasing popularity of artisanal products that often command a price premium. Home baking has seen an increase in popularity. A trend accompanied by the proliferation of artisanal bakeries in fashionable, ‘gentrified’ parts of UK cities. These trends are reported to be equally popular with younger consumers (under 25s) as with older generations – see MINTEL report on the UK Bread Market: https://store.mintel.com/report/uk-bread-market-report.

Despite these recent trends, the UK’s entrenched and enduring preference for refined grains necessitates the identification of foods in the UK diet most amenable to fibre-focused dietary change interventions. Since the cultural food landscape is likely more diverse in the UK than Denmark, owing to the greater ethnic diversity of the population and historical migration/colonial past, these interventions also need to acknowledge ethnic diversity in food preferences. Whole-grain intake at the UK population level is extremely low^(38–41)^ – particularly in low-socio-economic status (SES) groups^(40)^ – and there are few traditionally consumed whole-grain foods – such as Danish rye bread – that can be employed to anchor a campaign to re-establish traditional staple foods that have heritage value and imbued with nostalgic resonance. A partial exception was the popularity of Hovis bread whose 1970’s advertisements were steeped in nostalgia. The term ‘whole grain’ is not commonly employed in the UK; ‘wholewheat’, ‘brown’ and ‘wholemeal’ are preferred to define food products containing unrefined grains. These terms are arguably considered to be ‘worthy’ rather than ‘tasty’. Wholemeal is of particular interest since the term is protected by UK bread and flour regulations^(24)^ limiting the use of the term to products comprising only wholemeal flour. Despite this, breads using wholemeal labelling, yet comprising proportions of refined white flour, are on the market (https://www.sustainweb.org/news/jun22-warburtons-hovis-wholemeal-half-and-half-truth/). Consumer research suggests many consider high-fibre foods, particularly starchy foods, to be detrimental to health, associated with weight gain and digestive discomfort, reflected in the demand for gluten-free products far exceeding medical need^(25)^, a trend that prompted the formation of the DWP.

All UK age groups consume less than the recommended 30 g of fibre/d^(3)^. This shortfall appears stable: analysis of fibre intakes between 2008–2009 and 2016–2017 shows minimal change in levels of fibre consumed despite reformulation and public health campaigns^(42)^. The UK population obtains most of its dietary fibre from cereal and cereal products (∼40 % of total intake; predominantly breads, pastas, cereals) and fruit and vegetables (combined totals ∼30–40 %)^(5)^. There exist important differences across age categories. For example, breakfast cereals and fruit are greater sources of fibre in young children and over 65s compared with other age groups, whilst pasta, rice, pizza and meat/meat products predominate for adolescents compared with other age groups (see Table 2). Low intake of whole grain is likely to be contributing to inadequate fibre intake in the UK. However, data on UK whole-grain intake are limited. The median dry weight daily intake calculated from the 2008–2009 to 2010–2011 NDNS was 20 g/d for adults and 13 g/d for children and adolescents^(40)^. Whole-grain breads were the largest contributor to whole-grain intake (44 % in adults, 35 % in children/adolescents) followed by breakfast cereals (27 % in adults, 36 % in children/adolescents). Eighteen percentage of adults and 6 % of children and adolescents consumed no whole grain. Wheat (77 %) was the main whole grain consumed across all food categories. Oats accounted for 15 % of whole grain consumed, predominantly in the form of porridge (32 %) and ready to eat cereals (25 %)^(40)^.

**Table 2.** Percentage contribution of food groups to average UK daily fibre intake by age inclusive of National Diet and Nutrition Survey rolling programme years 9–11 (2016–2017 – 2018–2019). Main classified food group categories shown in bold. Adapted from the National Diet and Nutrition Survey. UK results from years 9–11 of the rolling programme (2016–2017 – 2018–2019). Available at: https://www.gov.uk/government/statistics/ndns-results-from-years-9-to-11–2016-to-2017-and-2018-to-2019 (Percentages)

Food group	Population age groups (years)					
	1·5–3	4–10	11–18	19–64	65–74	75+
	%	%	%	%	%	%
**Cereals and cereal products**	**40**	**41**	**44**	**38**	**37**	**42**
of which:						
* Pasta, rice, pizza and other miscellaneous cereals*	7	8	12	9	4	2
* White bread*	7	9	10	7	6	7
* Wholemeal bread*	3	3	3	5	6	7
* Brown, granary and wheat germ bread*	4	3	3	3	4	6
* Other breads*	0	0	0	1	0	0
* High-fibre breakfast cereals*	9	7	5	6	9	10
* Other breakfast cereals*	2	2	2	1	1	2
* Biscuits*	4	4	4	3	3	4
* Buns, cakes, pastries and fruit pies*	2	4	3	2	2	4
* Puddings*	1	1	1	0	1	1
** Milk and milk products**	**3**	**2**	**2**	**2**	**2**	**2**
** Eggs and egg dishes**	**0**	**0**	**0**	**1**	**1**	**0**
** Fat spreads**	**0**	**0**	**0**	**0**	**0**	**0**
** Meat and meat products**	**8**	**9**	**12**	**11**	**8**	**11**
** Fish and fish dishes**	**1**	**1**	**1**	**2**	**2**	**2**
** Vegetables and potatoes**	**22**	**26**	**25**	**30**	**32**	**27**
of which:						
* Salad and other raw vegetables*	1	2	2	3	3	2
* Vegetables (not raw) including vegetable dishes*	13	14	12	17	18	15
* Chips, fried and roast potatoes and potato products*	5	7	8	6	5	4
* Other potatoes, potato salads and dishes*	3	4	3	4	5	5
** Savoury snacks**	**2**	**3**	**3**	**2**	**0**	**1**
** Nuts and seeds**	**1**	**1**	**0**	**2**	**2**	**1**
** Fruit**	**16**	**11**	**6**	**8**	**11**	**10**
** Sugar, preserves and confectionery**	**2**	**3**	**2**	**2**	**1**	**1**
** Non-alcoholic beverages**	**1**	**1**	**1**	**1**	**0**	**0**
of which:						
* Fruit juice*	1	1	1	0	0	0
* Alcoholic beverages*	0	0	0	0	0	0
** Miscellaneous**	**4**	**3**	**3**	**3**	**4**	**4**
of which:						
* Dry weight beverages*	0	0	0	0	0	1
* Soup, manufactured/retail and homemade*	2	1	1	2	2	3
* Savoury sauces, pickles, gravies and condiments*	1	1	1	1	1	1
* Commercial toddler foods*	2	0	0	0	0	0
**Average daily fibre^†^ intake g**	**10·4**	**14·3**	**16·0**	**19·7**	**19·7**	**17·3**

† Fibre is measured by the American Association of Analytical Chemists (AOAC) methods. AOAC fibre includes resistant starch and lignin in the estimation of total fibre in addition to NSP.

One under-explored aspect of the success of the DWP is the association of whole-grain bread with particular models of masculinity where working class men have, for generations, been linked to high levels of fibre consumption, particularly rye bread, because of the dietary demands of farming, fishing and other manual occupations^(17)^. The same sex associations do not apply in the UK where the consumption of high-fibre foods is generally coded as female, as in popular commercials for Special K and other high-fibre breakfast cereals. Despite this, men have typically consumed more fibre in the UK compared with women^(5)^. There is some evidence that the food industry struggles to promote fibre as a desirable product feature. In 2018, Arla Foods launched a high-fibre yogurt enlisting the Wieden+Kennedy advertisement agency to produce a launch campaign that acknowledged that ‘traditional fibre-rich foods can be bland and uninspiring’^(43)^. The subsequent campaign focused heavily on fibre being boring and promoted the high-fibre yogurt products as a way to consume fibre without knowing or being able to taste the fibre. This ‘health by stealth’ approach contrasts markedly with the promotion of whole grain as a healthy, tasty and natural ingredient that characterises many of the DWP whole-grain promotion campaigns. Whole grains may be more appealing and simpler for consumers to comprehend than fibre, particularly in countries like Denmark with a historical connection with whole-grain foods. Whole grains are a food ingredient and are arguably easier to promote, and for consumers to envisage – more of the whole grain is retained and added to the food product that is associated with health benefits. Contrastingly, fibre as a broad nutrient may be less intuitively comprehendible.

Natural and healthy messages related to whole grain are likely more appealing to consumers compared with claims on fibre, which have focused on the authorised EFSA fibre claims related to stool transit and bulk, a difficult sell in any context, let alone in relation to food. However, there is limited evidence on UK consumer awareness and perception of whole grains compared with fibre, and how they would respond to a campaign on either. The Agriculture and Horticulture Development Board found that 75 % of consumers considered whole grain a healthy claim^(44)^. Whilst consumers are increasingly choosing food and drink for health^(45)^, it is uncertain how they perceive whole grain and how they would respond to messages on this compared with fibre.

## Ensuring equitable impacts

**Fig. 4.**
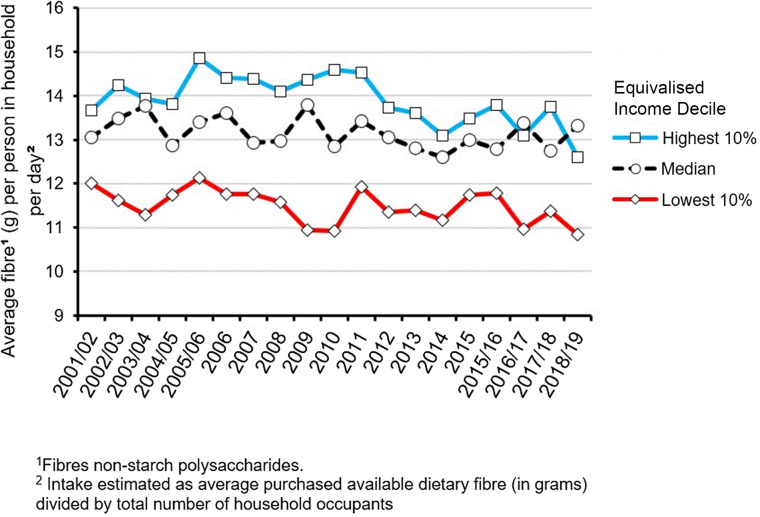
Average UK daily quantity of fibre purchased per person for highest, median and lowest equivalised income deciles 2001–2002 to 2018–2019. Taken from: DEFRA (2018–2019). Family food datasets: Equivalised income decile group, Household Nutrient Intakes: https://www.gov.uk/government/statistical-data-sets/family-food-datasets.

Dietary-related health disparities are unevenly distributed towards the lower end of the socio-economic gradient^(46)^. Furthermore, systematic reviews of health behaviour interventions among low-income populations have demonstrated a smaller positive effect size *v*. general populations^(47)^, suggesting that some dietary interventions may increase inequalities by disproportionately benefiting less disadvantaged groups (‘intervention-generated inequalities’). This highlights the need to tailor fibre interventions for low-SES groups as a method to reduce health inequalities. As such, it is critical that dietary interventions reach low-SES populations to help bridge the health inequality gap. The DWP recognised that significant increases in whole-grain intake were not evenly distributed across the socio-economic spectrum, with the lowest gains recorded in low-income households. As such, it is imperative that interventions designed to increase fibre or whole-grain consumption in the UK take necessary steps to ensure equitable distribution of benefit. This will be no easy feat since the causes of dietary-related health inequalities are complex and manifold. Household income is positively associated with greater consumption of nutritious foods and micronutrients^(5,48)^. In the UK, this holds true for the consumption of both whole grain^(40)^ and fibre in general^(5)^. Fig. 4 shows the average daily purchased fibre per household in the UK by selected equivalised income deciles. Whilst insufficient fibre availability has been shown across all income decile categories (< 30 g/d, SACN^(3)^), this is particularly inadequate in the lowest 10 % income decile. However, it is crucial to acknowledge the lower intake baseline the majority of low-income consumers are starting from when designing and evaluating interventions, both with regards to what is a feasible and sensible increase in fibre or whole grain intake and what success looks like in terms of relative increase from this lower baseline.

Many factors likely contribute to lower fibre intakes in lower-SES populations. The cost and affordability of food inevitably influence purchasing behaviour and dietary choice; impoverished circumstances lead to impoverished diets. The cost of healthier food is higher than unhealthy foods. Fruit and vegetables are often the most expensive food category; contrastingly, foods high in fat, sugar and/or salt are substantially cheaper^(49)^. In 2022, the average cost of healthier foods per 1000 kcal was estimated to be £8·51 compared with £3·25 for less healthy foods (calculated by average price of food and drink by Nutrient Profile Modelling score category^(49)^). The Food Foundation’s Broken Plate report shows that meeting the Government’s recommended Eatwell Guide would cost the poorest fifth of UK households 50 % of household disposable income, compared with 11 % in the richest fifth of households (calculated by income quintile^(49)^). The situation shows no sign of improving. Food prices are rising significantly, driven by global food system shocks^(50)^. Unprecedented inflation and a reduction in the real value of wages and state benefits have resulted in soaring costs for housing, energy and other essentials. As food is often the only flexible household expenditure, increased living costs inevitably reduce the amount that can be spent on food, ultimately reducing the quality of diets. Healthier foods, particularly whole-grain foods, are also marketed as premium foods and are often more expensive, or perceived to be so^(40,51)^. Cost is not the only barrier to low-SES households accessing healthier food, including whole-grain or high-fibre foods. The food environments in which people live directly affect accessibility to certain foods. Those living in deprived communities may face limited access to certain foods. For example, areas of high deprivation often have less access to fresh or ‘healthier’ foods both in supermarkets^(52,53)^ and due to a greater proliferation of fast-food takeaways^(49)^.

**Fig. 5.**
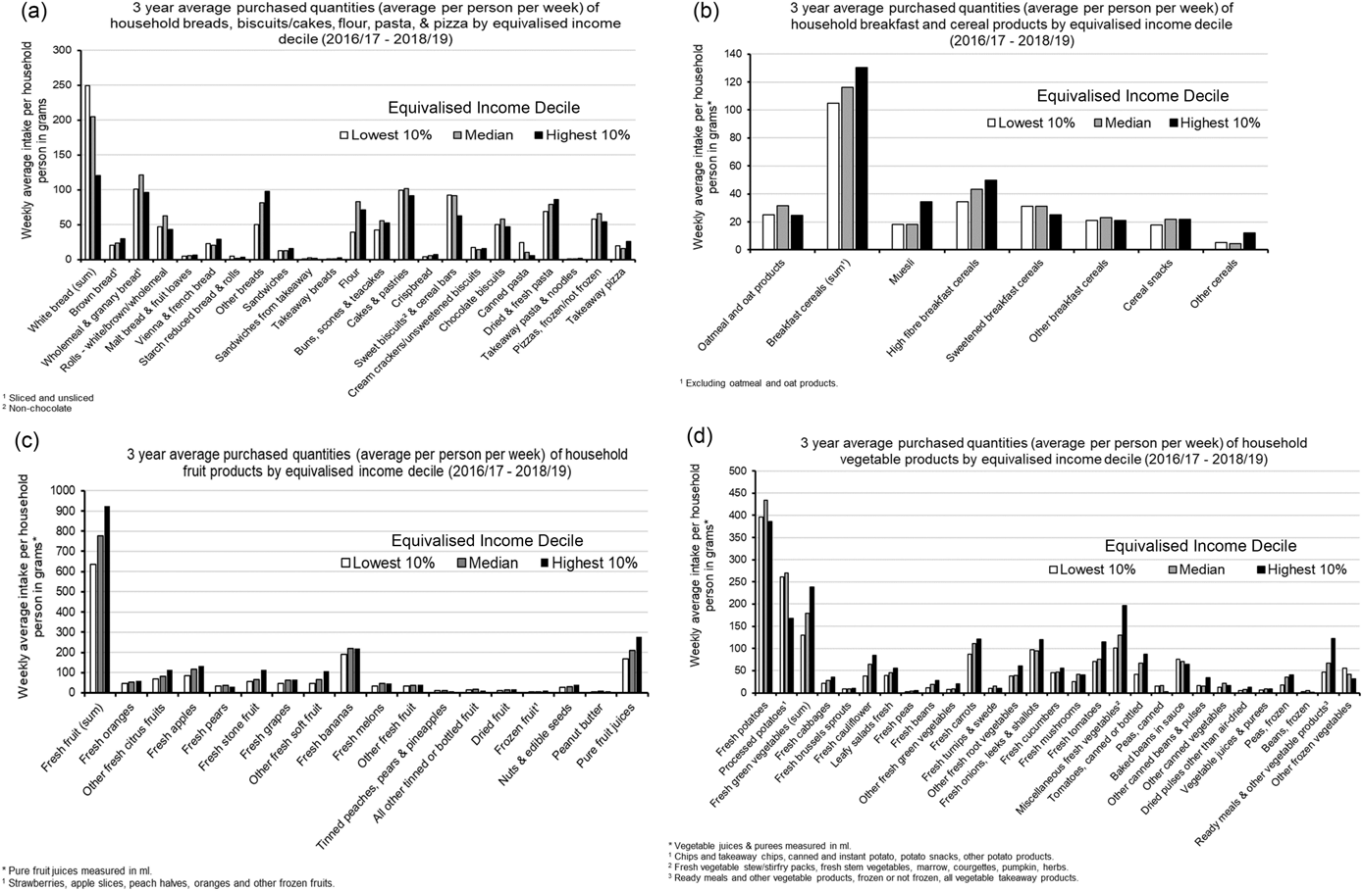
Purchased average household weekly quantities of: (a) breads, biscuits/cakes, flour, pasta and pizza; (b) breakfast and cereal products; (c) fruit products and (d) vegetable products, by lowest 10 %, median and highest 10 % equivalised income deciles. Figures represent 3-year average values inclusive of years 2016–2017 to 2018–2019). Amended from: DEFRA (2018–2019). Family food datasets: Equivalised income decile group, Household Nutrient Intakes: https://www.gov.uk/government/statistical-data-sets/family-food-datasets.

Greater insight is needed into the socio-cultural factors – for example, dietary preferences, cooking skills, food knowledge – influencing inadequate consumption of fibre and whole grain in lower SES populations. Understanding habitual dietary patterns and preferences is integral to designing equitable interventions to increase fibre/whole-grain intakes. Fig. 5 shows the average weekly quantities of food types purchased by UK households categorised by selected income decile (quantities shown comprise the 3-year average weekly household purchases between 2016/2017 and 2018–2019). Such data can be utilised to identify foods habitually consumed or lacking in lower-SES households. These consumption patterns may be reflective of the prohibitive costs of certain food types (e.g. the lowest 10 % income category consistently purchase less fresh fruit and vegetables) or socio-cultural preferences (e.g. the strong preference for white bread in the lowest 10 % income category). This information can inform the development of interventions to increase access to certain foods (e.g. fresh fruit and vegetables) or identify preferred foods suitable for reformulation to increase whole grain or fibre (e.g. increase the fibre content in specific bread varieties). Specific insights into the dietary patterns and preferences of black, Asian and mixed ethnicity households are also keenly needed since these ethnic groups are disproportionately affected by socio-economic disadvantage.

The mode of food access used to promote consumption of high-fibre or whole-grain foods is a further critical consideration if large-scale transformative change in dietary consumption is to be achieved. Specific targeting of food pathways and food environments with greater relevance for low-SES populations can ensure equitable benefits of improved fibre consumption are offered to those often most affected by dietary inequalities. Whilst the promotion of reformulated products on sale on the commercial market is a key factor in the success of the DWP, targeting institutional markets via so-called anchor institutions such as schools can also be utilised to reach a larger population. School-based interventions are a good way of promoting fibre consumption to a large population at a critical developmental stage. Childhood and adolescence are a period when dietary interventions could have a lasting impact, given that the health knowledge, values and behaviours that are developed during this life-stage are often embedded and track into adulthood. Interventions in schools can also deliver significant impact ‘at scale’ when targeted in areas of higher deprivation. Children living in low-income households are more likely to skip or consume poor dietary quality breakfast^(54,55)^ – a food category that is a key contributor of fibre and whole-grain intake in children and adolescents^(3,40)^. Therefore, increasing fibre or whole grain – via school breakfast programmes, for example – can make a significant and equitable contribution to intakes in children. Indeed, there is considerable evidence of the beneficial impacts of harnessing food provision within the school environment to increase children’s whole-grain^(56–62)^ and fibre^(63–65)^ intakes. Increasing the availability – for example, raising the proportion of whole-grain/high-fibre choices on offer and reformulating food to boost whole-grain/fibre content – and accessibility – particularly via increased eligibility to receive free school meal provision or via universal school breakfast programmes – can be powerful anchors to improve the diet quality of children that can be adopted in the UK.

### Conclusions

Wholesale transfer of the DWP model to the UK is considered unlikely given the absence of some of the key ‘success factors’ that were present in Denmark. These include Government backing at a national scale where the devolved nature of UK government and the complexity of the regulatory environment work against a united approach. The lack of clear definition and regulation of whole grain – with an agreed knowledge base and accepted logo – also differentiate the Danish from the UK experience. The UK is currently misaligned with many nations on whole grain including Canada, Denmark, Australia and the USA, all of which have specific dietary guidelines on whole-grain intake. Increasing the availability of whole grain was also central to the success of the DWP and similar approaches are necessary in the UK, ideally in commonly consumed and accessible food forms. To facilitate this, the UK needs a standardised definition of whole grain and a recommended whole-grain intake amount to incentivise reformulation and introduction of whole-grain products to market. The dietary benefits of whole grain also need greater promotion to increase public awareness of the need to consume whole-grain products.

In the absence of whole-grain regulation and dietary recommendations, prioritisation of fibre intake may be a more efficacious approach to increasing dietary quality in the UK. The 2021 National Food Strategy for England identified increased consumption of fibre as one of four key population dietary shifts needed in the UK. However, the lack of recognition of fibre in the Government’s Food Strategy for England suggests fibre is not high on the current food agenda. The strategy does acknowledge the need for ‘government and industry working in partnership on a shared endeavour to promote healthier diets’. Such public–private partnerships, exemplified by the DWP, are a promising policy tool to facilitate ‘cross-sector’ working towards a common goal that can be used to help achieve dietary goals in populations. Initiatives within the food industry should be supported and Government must engage with industry to ensure the reformulation and development of high-fibre products are promoted and incentivised by clear regulation and supported by public engagement to communicate the health benefits of fibre intake. Collaborative work across the devolved nations is also crucial to ensure a consistent and clear approach.

It is imperative that targeted measures are employed to ensure the promotion of increased fibre and whole-grain intakes are equitably distributed across the UK population. Since diet quality and food insecurity are intimately linked, more needs to be done to ensure low-SES households are able to access higher quality diets both in terms of income available to purchase food and food environments that permit access to nutritious food. Further, the preferences, socio-cultural factors and food environments most relevant to low-SES communities must be identified and utilised in the promotion of whole grain and fibre.

Whilst the wholesale transfer of the DWP approach to the UK is considered unrealistic owing to the identified geographic, political, regulatory and socio-cultural factors, the DWP approach and success factors provide an invaluable template against which the key barriers and deficiencies in the UK context affecting population level whole grain/fibre dietary change can be identified. Upon this foundation, intervention methodologies and policy recommendations can be put forward to better position the UK to start replicating some of the successes of the DWP.

## Data Availability

Data sharing is not applicable to this article as no new data were created or analyzed in this study.

## Footnotes

1 Recommended dietary fibre intakes are adjusted for children based on age: aged 2–5 years = approximate 15 g/d; aged 5–11 years = 20 g/d; aged 11–16 years = 25 g/d; aged 16–18 years = 30 g/d.
